# A Delayed Mental Hospital Appointment

**Published:** 1913-06-07

**Authors:** 


*To the Editor of* THE HOSPITAL.

### A Delayed Mental Hospital Appointment.

*To the Editor of* THE HOSPITAL.

SIR,—I find in this week's issue of your journal that you notice that "a peculiar position has arisen at the North Wales County Asylum at Denbigh with regard to the appointment of a medical superintendent."

The situation is indeed peculiar, and much more so to those who know how great the competition is for the medical superintendency of a county asylum. For such positions there are usually over sixty applications from men of eight to sixteen years' asylum experience, who have been actually carrying out the duties of deputy medical superintendents, and who, consequently, have the administrative experience usually (out of North Wales) considered indispensable. They comprise many men of excellent qualifications and talents, many of whom have had hospital and other experience before definitely devoting themselves to asylum work, and who quite expect to have to give at least eight years of close study to their speciality, and especially have several years of doing the largely administrative work of a first assistant medical officer and deputy medical superintendent before they have a fighting chance of a superintendency.

What, then, is the reason that there were only nine applications and that the three of these that were put on the short list comprised: (1) An assistant medical officer of health who has been a few months in an asylum and who qualified in 1907 (his name is unmistakably Welsh); (2) the fourth assistant in an asylum, a man who qualified in 1908 (his name is also Welsh); (3) the third assistant in an asylum (his name is also Welsh)?

Is the answer set forth in the advertisement of the appointment that candidates must have a knowledge of colloquial Welsh?

It is obvious, however, that this provision merely masks the intention of the committee to get a Welshman, no matter what his experience may be, as there are several deputy medical superintendents in Welsh asylums who are sure to have applied, but who, unfortunately, do not rejoice in the possession of unmistakably Welsh names, though carrying out their duties to their patients successfully. But excluding these few deputy medical superintendents in Welsh asylums, why would not a deputy medical superintendent from an English asylum do?

It is found in the various Government Medical Services —*e.g.*, Colonial Medical Service, Egyptian Quarantine Service, Soudan Medical Service, Medical Department of Chinese Customs Service—that the average British medical man, even with the handicap of being English or Irish, can in the early days of his appointment secure a degree of proficiency in the colloquial adequate to the efficient performance of his duties.

Would, then, the average deputy medical superintendent of an English asylum be less resourceful if he got a superintendency in Wales? I think not. That the appointment was postponed shows that there must have been some little recognition of the nature of the competition and that the whole committee were not imbued with narrow-minded parochialism.

In this connection it is to be noticed that the age-limit in the first advertisement was "not over 45," and this is usually considered latitude enough, so that this can hardly be the real reason of postponement. It might be drawn to the notice of the committee that in Ireland, where they have the reputation of being much less exacting than in England over qualifications for appointments generally, the Privy Council rules prevent anyone being appointed a medical superintendent who has not been qualified seven years and also had five years' experience in an asylum.

I have no personal knowledge of the three men on the short list, and quite credit that for their length of service and opportunities they are excellent men, and I have only spoken of them in comparison with their seniors in English and Welsh asylums. The kernel of the subject is that just at the present time there is no Welshman with the standard administrative qualifications for the post, and that, rather than give it to one who was not a Welshman, the committee would disregard these qualifications entirely.

Finally, I may say that I know no instance of Welsh candidates for appointments in England being discriminated against.—Yours faithfully,

AN ASYLUM MEDICAL OFFICER.

June 2, 1913.

